# Living alone and provider behaviour in public and private hospitals

**DOI:** 10.1016/j.jhealeco.2025.103016

**Published:** 2025-08

**Authors:** Luigi Siciliani, Jinglin Wen, James Gaughan

**Affiliations:** a Department of Economics and Related Studies, University of York; Economics of Health and Social Care Research Unit (ESHCRU), United Kingdom; b Centre for Health Economics, University of York, United Kingdom

**Keywords:** Public and private providers, Health care, Living alone

## Abstract

Following COVID-19, hospitals in many OECD countries are under pressure to absorb backlogs accumulated due to the suspension of health services. Reductions in length of stay can generate capacity to treat patients and increase efficiency. Personal circumstances, such as living alone, can affect how long patients stay in hospital. We test whether such non-clinical factors affect care received by patients. Several countries are experiencing an increase in the number of elderly people who live alone. Patients who live alone may lack support at home leading to delayed discharges despite being clinically fit. We test whether living alone affects length of stay of publicly-funded patients treated by public and private hospitals requiring hip replacement, a common planned surgery, in England. Private providers have stronger incentives to contain costs, which could reduce the extent to which non-clinical factors such as living alone are taken into account when providers discharge patients. Using administrative data and controlling for a rich set of patient characteristics, and hospital and local supply factors, we provide evidence that living alone increases length of stay. The effect is substantive and larger for public hospitals and older patients. It is similar for patients living in urban and rural areas, and across socioeconomic status. More broadly, the study shows that non-clinical factors can affect the care received by patients.

## Introduction

Following the COVID-19 pandemic, hospitals are under pressure to absorb elective backlogs that have accumulated due to the suspension of health services (OECD, 2023). Given limited supply of hospital beds, an ageing population and increasing shortages of health personnel, reductions in length of stay can generate additional capacity to treat patients and increase efficiency. Due to changes in demographics, life expectancy and cultural norms, several countries are experiencing an increase in the number of elderly people who live alone. 30% of people over 65 years old lived alone across the OECD countries in 2022, and 37% in the United Kingdom (OECD, 2024).

Personal circumstances and living arrangements, such as living alone, can affect how long patients stay in hospital. Patients who live alone may lack support at home once discharged and lack the help with activities of daily living. As a result, doctors who become aware of such personal circumstances may be reluctant to discharge the patient quickly and instead decide to keep the patient longer despite being clinically fit for discharge. On the other hand, patients living alone could have other sources of support (e.g. family and friends) that mitigate such effects. The extent to which living alone affects length of stay is therefore in principle indeterminate. If non-clinical determinants, such as living alone, affect length of stay there is scope for implementing policies that support hospitals to discharge patients when clinically ready to be discharged.

This study tests if living alone affects length of stay in public and private hospitals treating publicly-funded (National Health Service) patients in England. In broader terms, our study tests whether non-clinical factors affect the amount of care that patients receive. We focus on patients who require an elective hip replacement, which is a common non-emergency surgery. Like other OECD countries, such as France, Italy and Germany, publicly-funded patients are treated by a mix of public and private providers (Siciliani et al., 2022). In England, about 25% of patients were treated in 2016 by private hospitals (known in England as the Independent Sector), which increased to 37% in 2021. Entry by private providers was facilitated from 2003 onwards to increase capacity and stimulate competition (Moscelli et al., 2021).

Economic theory suggests that private providers have stronger incentives to contain costs to maximise profits (Glaeser and Shleifer, 2001; Brekke et al., 2012; Brekke et al., 2015). This could translate into a shorter length of stay and affect the extent to which non-clinical determinants, such as living alone, are considered. Driven by a stronger profit motive, private providers could insist that the patient is discharged once clinically ready regardless of their personal circumstances. Public providers instead could give a higher weight to patients’ health and utility, therefore also including non-clinical circumstances such as living alone (Brekke et al., 2011).

After setting out a simple theoretical framework, we use administrative data from the Hospital Episode Statistics matched with Patient-Reported Outcome Measures Survey (PROMs) before (2016/17-2019/20) and after COVID-19 (2020/21-2022/23). Health systems had to respond to new challenges during the pandemic, which has affected their organisation and provision of care. We therefore test the effect of living alone on length of stay separately before and after COVID-19.

To identify the effect of living alone, we estimate patient-level linear regression models and control for a rich set of patient characteristics that could correlate both with length of stay and the probability of living alone. For example, older patients are both more likely to have a longer length of stay and to live alone. Patients in worse health have a longer length of stay and health status could correlate (positively or negatively) with the probability of living alone. We control for pre-operative health (measured by a hip specific clinical indicator), age, ethnicity, primary and secondary diagnosis, comorbidities and past emergency admissions. Some hospitals are more efficient than others, which translates into a shorter length of stay, and located in areas with a higher or lower proportion of patients living alone. We therefore control for systematic differences in length of stay across providers by adding hospital (or specialist) fixed effects. Last, hospital ability to discharge patients promptly could depend on the availability of local supply of care homes, rehabilitation and community care. To control for these factors, we include small area fixed effects, known as Lower Super Output Areas (LSOAs). LSOAs are small statistical units constructed from the national census with a mean population of about 1,500 inhabitants. We therefore exploit variation in whether patients live alone within hospitals and within the same small geographical area, while controlling for a rich set of patient characteristics that relate to their health status.

Private providers tend to treat less complex patients. To test the extent to which differences in the effect of living alone on length of stay between public and private providers is due to the different casemix, we use (coarsened exact) matching techniques. We match on a range of patient characteristics, including pre-operative health measured by clinically validated indicators (such as the Oxford Hip Score).

We find that patients who live alone stay 0.23 days longer in private hospitals and 0.42 days longer in public hospitals in the pre-pandemic period. They stay 0.17 days longer in private hospitals and 0.39 days longer in public hospitals following the pandemic. After matching to control for casemix, the effect of living alone in public hospitals reduces from 0.42 to 0.36 days in the pre-pandemic period and from 0.39 to 0.32 days following the pandemic. We also show that the effect of living alone is concentrated in the post-operative rather than the pre-operative length of stay. Our results are not due to patients who live alone having to wait for a place in a care home or a rehabilitation centre: we show that there are very few patients who do not return to their usual place of residence after the surgery. The effect is also not due to patients living alone being in worse pre-operative health. Patients who live alone tend to have better pre-operative health, and omitting pre-operative health reduces the effect of living alone on length of stay.

Heterogeneity analysis shows that the effect is larger for older patients. In the pre-pandemic period, patients who live alone and are under 70 years old stay 0.15 days longer in private hospitals and 0.34 days longer in public hospitals. Instead, for patients aged over 70, the effect of living alone increases to 0.36 days for private hospitals and to 0.64 days in public hospitals. The effect of living alone on length of stay is relatively similar for male and female patients for private hospitals (0.25 vs 0.24 days), and higher for male in public hospitals (0.46 vs 0.40 days). The effect is similar for patients living in urban and rural areas. The effect of living alone is a little higher for patients who live in areas whose deprivation is above the median in private hospitals (0.24 days vs 0.22 days) but is similar in public hospitals.

Our study relates to different strands of the literature. First, our study relates to the economics literature testing for differences in provider behaviour between public and private providers. The literature in the health sector has focused on differences in quality and efficiency (Shen, 2002; Lien et al., 2008; Herr et al., 2011; Moscelli et al., 2018; Dalton and Bradford, 2019). We are not aware of any studies that focus on differences in how non-clinical determinants, such as living alone, affect length of stay. Turner et al. (2016) investigates the association between living alone and a range of health outcomes (waiting times, symptom duration, risk of admission and complications, and length of stay) in 2009/2011 but does not differentiate between public and private providers. In terms of methods, we also include hospital and small area fixed effects to control for a range of supply factors both at the hospital level and the local supply of care homes, rehabilitation and community care. Moreover, we look at differences in the effect before and after COVID-19 that has significantly put health systems under strain, and across patient groups. In our sample period, the involvement of private providers has increased over time, which motivates our matching analysis.

Second, our study relates to the literature on bed blocking and the effect of availability of long-term care on delayed discharges. Gaughan et al. (2015) show that higher availability of care home beds in England reduces delayed discharges. Moura (2022) finds that the entry of home care in Portugal reduces bed-blocking among patients with high care needs. Fernandez et al. (2018) find that a higher number of local authorities involved in care planning and commissioning of social care services for discharges increases hospital lengths of stay likely due to lack of coordination. Third, our study relates to the literature on the effect of informal carers on the utilisation of health services. Van Houtven and Norton (2008) find that informal care by children reduces Medicare long-term care and inpatient expenditures of single elderly, but less so for those who are married. Bolin et al. (2008) find that while informal care is a substitute of formal home care, it is a complement to doctor and hospital visits. Last, some studies focus specifically on the effect of living alone on healthcare utilisation. Using data from one large hospital in Italy, Agosti et al. (2018) show that living alone was significantly associated with a longer hospital stay. Using administrative data from Switzerland for inpatients with chronic conditions, Bayer-Oglesby et al. (2022) find that living alone is associated with longer length of stay, though partially mediated by the number and type of comorbidities. A more substantive literature focuses on the effect of living alone and loneliness on physical and mental health with mixed findings (Turner et al., 2016; Tamminen et al., 2019; Zhao et al., 2022; Fawaz and Mira, 2023).

## Theoretical framework

To fix ideas, we develop a simple model of provider behaviour. In line with previous studies (Ellis and McGuire, 1986; Chalkley and Malcomson, 1998), we assume that a representative provider maximises a weighted sum of patient benefit and provider profit. Providers can be public or private. We denote provider status with *i={PU,PR}*, where *PU* stands for public and *PR* for private. We define $V^{i}\left(l,a\right)$ as provider utility from treating a patient with a length of stay *l* who has living arrangement *a* (equal to 1 if living alone and equal to zero if living with a spouse, partner or family). Patient utility from hospitalisation is given by h(l,a)+u(l,a), where h(l,a) is the health benefit which increases with length of stay up to a level $l^{h}$ and reduces with length of stay for higher levels (e.g. due to risks of hospital acquired infections); u(l,a) includes non-health dimensions of patient utility from length of stay, e.g. from help in daily activities, such as food, getting dressed and being taken care of. Again, we assume that this utility component increases with length of stay up to $l^{u}$ and decreases afterwards (e.g. due to patient desire to return to their own home). We allow for living alone to affect both components of patient utility function, as discussed in more detail below.

We assume provider utility to be additively separable in the profit and the altruistic component:

$$V^{i}\left(l\right)=\alpha^{i}\;\left[h\left(l,a\right)+\gamma^{i}\;u\left(l,a\right)\right]+\delta^{i}\;\left[p-c\left(l\right)\right],$$

where $\alpha^{i}$ is the degree of provider altruism or intrinsic motivation. $\delta^{i}$ is the weight that the provider gives to profit. *p* is the Diagnosis Related Group (DRG) tariff and c(l) is provider cost, which is assumed to be increasing in length of stay $\left(c_{l}>0\right)$. $\gamma^{i}$ is the weight that the provider gives to the non-health component of patient utility. It could be argued the healthcare providers care more about the health of the patient, which is in line with their main mission, than other dimensions of patient experience, which would imply $\gamma^{i}<1.$

The optimal length of stay, denoted with $l^{i*}$, chosen by the provider is such that $V_{l}^{i}\left(l^{i*}\right)=0$ or, more extensively:

$$\alpha^{i}\;\left(h_{l}+\gamma^{i}\;u_{l}\right)=\delta^{i}\;c_{l}\;.$$

The length of stay is determined such that the benefit from a marginal increase in length of stay (either higher health benefit or patient utility) is equal to the marginal cost. We assume that we have an interior solution, and the problem is well behaved, so that $V_{ll}^{i}=\alpha^{i}\;\left(h_{ll}+\gamma^{i}\;u_{ll}\right)-\delta^{i}\;c_{ll}<0.$ We assume that private providers give a higher weight to profits and marginal costs, $\delta^{PR}>\delta^{PU}$, which reduces length of stay (see Appendix). This effect is further reinforced if private providers attract less altruistic doctors, $\alpha^{PR}<\alpha^{PU},$ and give lower weight to patient utility, $\gamma^{PR}<\gamma^{PU}$ (see Appendix).

We assume that, for a given length of stay, patients who live alone have a higher marginal utility from length of stay so that $u_{l}\left(l,a=1\right)>u_{l}\left(l,a=0\right)$. This is because patients who live alone have less support at home and find it more difficult to take care of themselves and to perform the daily tasks of living. Patients who live alone may also struggle more to do rehabilitation at home without support which could reduce their health benefit and increase the marginal health benefit from staying longer in the hospital, $h_{l}\left(l,a=1\right)>h_{l}\left(l,a=0\right)$. For a given level of altruism and profit weight, patients who live alone have a longer length of stay as long as living alone increases the marginal health or utility from length of stay (see Appendix). This effect is larger, the higher is the weight that the provider gives to the non-health component of patient utility. We therefore have that both public and private hospitals have a longer length of stay for patients who live alone, $l^{*PU}\left(a=1\right)>l^{*PU}\left(a=0\right)$ and $l^{*PR}\left(a=1\right)>l^{*PR}\left(a=0\right)$. If instead living alone does not affect the health effect from a marginal increase in length of stay and if providers give zero weight to the non-health component of patients utility function, then living alone will have no effect on length of stay.

Whether the difference is length of stay between those who live alone and those who do not live alone is higher or lower for public providers or private providers depends on two effects. A higher weight on profits by private providers implies a higher marginal cost which reduces length of stay, and also dampens the differential provider response between those living alone and those who do not live alone (because the cost function is effectively more convex for private providers). Second, if the marginal health and utility from length of stay decreases more quickly with length of stay for patients living alone than those not living alone then this effect tends to increase the difference in length of stay between those living alone and those not living alone for private providers (see Appendix). If the first effect dominates then $l^{*PU}\left(a=1\right)-l^{*PU}\left(a=0\right)>l^{*PR}\left(a=1\right)-l^{*PR}\left(a=0\right).$

## Institutional background

The National Health Service (NHS) in England is publicly funded. It provides universal access to health care, which is free at the point of use and allocated based on need and not ability to pay. Primary care doctors (General Practitioners, GPs) act as gatekeepers and patients have to visit a GP to obtain a referral to a hospital specialist. This applies also to patients with symptoms of hip osteoarthritis, who are then referred to a hospital specialist in the orthopaedics department. Subsequent hospital visits involve an assessment of patient condition and diagnostic tests such as X-rays or Magnetic Resonance Imaging (MRI) scans to determine the need for surgery. Once deemed ready for surgery, patients are added to a waiting list until they are booked to undergo the procedure as inpatients at the hospital.

Publicly-funded patients can be treated in public hospitals (NHS Trusts) or in private hospitals (the Independent Sector). Most private providers have for profit status. After the introduction of policies in 2002/3 that promoted patient choice of provider, the proportion of all planned patients treated in private providers increased from 2% in 2006 to 4.5% in 2013. The increase was even larger for certain non-emergency high-volume procedures. The proportion of publicly-funded hip replacements carried out in private providers increased from 3% in 2006 to 18% in 2011 and further to 25% in 2020 (Moscelli et al., 2018). Until 2019, hospitals were not reimbursed for re-admissions that arise within 30 days from discharge. The policy applied to both public and private providers, but was abolished after 2019 (NHS England, 2022; Department of Health, 2014; HFMA, 2011).

## Data

This study uses patient-level data from the Hospital Episodes Statistics (HES) for all planned (i.e. non-emergency) hip replacement surgeries in England performed from April 2016 to March 2023. Our data contain demographic, medical and administrative information about all NHS-funded care provided. Given its breadth of clinical information, its near-universal coverage and its longitudinal function, it has been used extensively by the research community (Boyd et al., 2017).

We match these data to information extracted from the Patient Reported Outcome Measures (PROMs) Programme via the unique patient identifier. Since April 2009, a national PROMs programme has been launched to collect information from patients themselves about their health (NHS England, 2018). All NHS-funded hip and knee replacement patients are invited to participate in surveys before and after surgery through a paper-based questionnaire. PROMs differ from general patient experience surveys and focus on patient health status, functioning and health-related quality of life. Health is measured both before and after the surgery, which allows to compute the health gain.

Across the study period, there are around 480,000 surgeries in the HES admitted patient dataset. Around 50% of all hip replacement patients are successfully linked with the PROM record, which corresponds to approximately 240,000 admission records.^1^ This is mostly due to patients not filling out the questionnaire.

### Independent variable

We use data from the PROMs on the living arrangement as the key independent variable. Patients are asked about their living arrangement: “Which statement best describes your living arrangements? 1) I live with a partner/spouse/family/friends; 2) I live alone; 3) I live in a nursing home, hospital or other long-term care home; 4) Other”. Around 75% and 25% of patients live with partner/spouse/family/friends and live alone before the surgery, respectively in our sample. We exclude the rest of patients with other living arrangements, which count for less than 1% of the sample.^2^ Our independent variable is a binary variable that equals 1 if the patient lives alone and 0 if the patient lives with partner/spouse/family/friends.

### Dependent variables

Our main outcome of interest is patients’ length of stay (LOS), which is defined as the difference in days between the date the patient was discharged from hospital, and the date they were admitted. We further decompose LOS for hip replacements into pre- and post-operative LOS (Cooper et al., 2018): the period from admission to surgical operation (pre-operative LOS), and the period from surgical operation to discharge (post-operative LOS). Pre-operative LOS has been used as a proxy of efficiency of health care (Cooper et al., 2018). In addition, we also examine the association between living arrangement and post-operative (and pre-operative) health status. To measure it, we extract data on Oxford Hip Score from PROMs, which consists of a total of 12 items. It is a validated questionnaire specifically to assess mobility status and pain for hip replacement surgery patients, ranging from 0 (worst) to 48 (best).^3^

### Other variables

We have data on socioeconomic and demographic characteristics of patients that are used as control variables: pre-operative Oxford Hip Score, age, sex, ethnicity, chronic conditions, the length of symptoms, the number of Elixhauser comorbidities, and hospital admission experience one year prior to the admission for hip replacement. We also take account of the corresponding lower-level super output area (LSOA), healthcare resource groups (HRGs) and the consultant who is directly involved in the delivery of the patients direct care. Furthermore, we use information on the index of multiple deprivation (IMD) and rurality to conduct heterogeneity analysis.

After sample cleaning and restriction to linked PROMs records, our final sample includes 219,645 patients for the main analysis. We split the sample between the pre-covid period, which includes financial years 2016/17 to 2019/20, and the post-covid period, which includes financial years 2020/21 to 2022/23 (recall that the financial year starts in April). This gives a sample 158,968 observations for pre-covid period and 60,677 observations for post-covid period)^4^. As a secondary focus, we conduct the analysis for post-operative health where the sample size is 153,711, as some patients do not complete the follow-up survey after surgery (122,979 for pre-covid period and 30,732 for post-covid period).

## Methods

We study the effect of living alone on length of stay for hip replacement surgery using the following linear regression model:

(1) $$LOS_{ihj}=\;\beta \;Alone_{i}\;+\;X_{i}^{\prime }\gamma +\;d_{h}+\;d_{j}+\varepsilon_{ihj}\;$$

where *LOS_ihj_* is the length of stay (measured in days) for patient *i* in hospital *h* and in LSOA *j*. Our main focus is on the length of stay. *Alone_i_* is an indicator of living arrangements that takes the value 1 if the patient lives alone, and 0 otherwise. *X_i_* is a vector of patient characteristics to control for patient casemix. These include age, sex, ethnicity, pre-operative Oxford Hip Score, chronic conditions, Healthcare Resource Groups (HRGs), symptom length, number of Elixhauser comorbidities and whether the patient had a hospital admission in the year prior to the admission for hip replacement.

*d_h_* is a vector of hospital fixed effects to control for systematic differences in LOS across hospitals, and *d_j_* is a vector of LSOA fixed effects to control for local supply differences in the availability of care homes, rehabilitation etc*.* ε*_ihj_* is the error term. The key coefficient of interest is *β* that captures the effect of living alone on length of stay. In addition to length of stay, we also examine the effect of living alone on post-operative health (measured by the post-surgical Oxford Hip Score). We estimate (1) by Ordinary Least Squares.

We are also interested in identifying whether the effect of living alone differs between public and private hospitals. Therefore, we estimate the following equation:

(2) $$\begin{array}{c} \mathrm{LO}S_{\mathrm{ihj}}\;=\;\alpha_{1}\;\mathrm{Alon}e_{i}\;+\alpha_{2}\;\left(\mathrm{Alon}e_{i}\;\times \;\mathrm{Publi}c_{i}\right)+\;\alpha_{3}\;\mathrm{Publi}c_{i}\;+\;X_{i}^{\prime }\;\theta_{1}+\;\left(X_{i}^{\prime }\;\times \;\mathrm{Publi}c_{i}\right)\;\theta_{2}+d_{h}\;+\;d_{j}\;+\varepsilon_{\mathrm{ihj}} \end{array}$$

where *Public_i_* is a binary indicator equal to 1 if patient *i* is treated by a public hospital and 0 if treated by a private hospital. Similarly to eq. (1), *Alone_i_* is an indicator variable equal to 1 if the patient lives alone, and 0 otherwise; *X_i_* is a vector of patient characteristics, *d_h_* is a vector of hospital fixed effects, *d_j_* is a vector of LSOA fixed effects, and ε*_ihj_* is the error term.

*α_1_* estimates the impact of living alone on the length of stay of patients treated by private hospitals, while the sum of *α_1_* and *α_2_* estimate the impact of living alone for patients treated by public hospitals. *α_2_* estimates the difference in the effect of living alone between public and private hospitals, while *α_3_* estimates the differences in length of stay between public and private hospitals (for patients not living alone). We adopt a flexible specification and allow for the effect of patient characteristics to vary between public and private hospitals (captured by the term ($X_{i}^{\prime }$ *× Public_i_*)).

One possible concern in comparing public and private hospitals is that patient casemix may differ and that private hospitals may treat less complex patients, a form of cream skimming. To further address this concern, we replicate the analysis but match patients from private hospitals with patients from public hospitals. Specifically, we implement Coarsened Exact Matching (CEM) to reduce imbalance in patient casemix between public and private hospitals.^5^

We implement the following matching approach. First, we select a set of patient characteristics for matching. These include pre-surgical age, sex, ethnicity, Oxford Hip Score, chronic conditions, symptom length, the number of Elixhauser comorbidities, and hospital admission one year prior to the admission for hip replacement. Second, given that we have a large sample of patients treated in public and private hospitals we implement k2k matching where every patient treated by a private hospital is matched with one patient treated by a public hospital. Last, we re-run the regression analysis for the matched sample.

## Results

Table 1 reports the descriptive statistics before and after the pandemic. The average hospital length of stay was 3.33 days in the pre-covid period and reduced to 2.67 days post-covid. There are reductions in both pre-surgical and post-surgical length of stays (from 0.05 to 0.02 and from 3.29 to 2.64, respectively). Around 65% of patients have a pre-operative OHS between 9 and 20 points and the average post-operative OHS is 40.25 points before COVID-19. The patient composition remains relatively stable in the COVID-19 period. Living arrangements are such that 25 percent of patients live alone while 75 percent live with a partner, spouse, family or friends before and after the pandemic. Table 2 shows that length of stay is systematically longer in public hospitals than in private hospitals. On average, length of stay was 3.6 days in public hospitals and 2.82 days in private hospitals (and 2.97 days versus 2.19 days in the COVID-19 period; see Table 2).

**Table 1:** Summary statistics.

*2016-2019*					*2020-2023*			
	Mean	S.D.	Min	Max	Mean	S.D.	Min	Max
Length of Stay (days)	3.33	1.81	0	13	2.67	1.72	0	13
Pre-operative	0.05	0.36	0	13	0.02	0.18	0	8
Post-operative	3.29	1.82	0	13	2.64	1.72	0	13
Pre-operative Oxford Hip Score (OHS)								
0 to 8	0.15	0.36	0	1	0.17	0.38	0	1
9 to 16	0.34	0.48	0	1	0.35	0.48	0	1
17 to 24	0.31	0.46	0	1	0.30	0.46	0	1
25 to 32	0.20	0.37	0	1	0.14	0.35	0	1
Over 33	0.04	0.20	0	1	0.04	0.19	0	1
Post-operative OHS	40.25	8.35	0	48	40.09	8.64	0	48
Live with Spouse	0.75	0.43	0	1	0.75	0.43	0	1
Live Alone	0.25	0.43	0	1	0.25	0.43	0	1
Public Hospital	0.66	0.47	0	1	0.60	0.49	0	1
*Age Band*								
Under 30	0.00	0.06	0	1	0.00	0.06	0	1
[30, 60)	0.21	0.41	0	1	0.22	0.41	0	1
[60, 65)	0.13	0.33	0	1	0.14	0.35	0	1
[65, 70)	0.17	0.38	0	1	0.16	0.37	0	1
[70, 75)	0.20	0.40	0	1	0.19	0.39	0	1
[75, 80)	0.15	0.36	0	1	0.16	0.37	0	1
Over 80	0.13	0.34	0	1	0.13	0.33	0	1
Male	0.40	0.49	0	1	0.40	0.49	0	1
White	0.83	0.38	0	1	0.74	0.44	0	1
Other Ethnicity	0.00	0.05	0	1	0.00	0.06	0	1
Unknown Ethnicity	0.16	0.36	0	1	0.24	0.43	0	1
*Chronic Conditions*								
Heart Disease	0.08	0.27	0	1	0.08	0.27	0	1
High Blood Pressure	0.37	0.48	0	1	0.36	0.48	0	1
Stroke	0.01	0.11	0	1	0.01	0.11	0	1
Circulation	0.05	0.21	0	1	0.04	0.20	0	1
Lung Disease	0.08	0.28	0	1	0.08	0.28	0	1
Diabetes	0.09	0.29	0	1	0.09	0.28	0	1
Kidney Disease	0.02	0.14	0	1	0.02	0.14	0	1
Nervous Symptom	0.01	0.09	0	1	0.01	0.09	0	1
Liver Disease	0.01	0.08	0	1	0.01	0.09	0	1
Cancer	0.05	0.23	0	1	0.06	0.23	0	1
Depression	0.10	0.30	0	1	0.11	0.31	0	1
Arthritis	0.73	0.45	0	1	0.72	0.45	0	1
*Symptom Length*								
Under 1 Year	0.11	0.32	0	1	0.06	0.24	0	1
1 to 5 Years	0.69	0.46	0	1	0.73	0.44	0	1
6 to 10 Years	0.12	0.33	0	1	0.13	0.34	0	1
Over 10 Years	0.07	0.25	0	1	0.07	0.26	0	1
Elixhauser Index	1.39	1.24	0	10	1.50	1.31	0	10
*Past Admission Last Year*								
No Admission	0.64	0.48	0	1	0.68	0.47	0	1
One Admission	0.23	0.43	0	1	0.21	0.41	0	1
Two Admissions	0.08	0.27	0	1	0.07	0.25	0	1
Three Admissions	0.03	0.16	0	1	0.02	0.14	0	1
Over Three Admissions	0.02	0.14	0	1	0.02	0.14	0	1
*Past Emergency Admission Last Year*								
No Admission	0.92	0.27	0	1	0.92	0.27	0	1
One Admission	0.06	0.24	0	1	0.06	0.24	0	1
Two Admissions	0.01	0.10	0	1	0.01	0.11	0	1
Over Two Admissions	0.00	0.04	0	1	0.00	0.04	0	1

Note: the sample size for variables before Covid is 158,968 (except for the post-operative Oxford Hip Score, which is 122,979), while the sample size after Covid is 60,677 (except post-operative OHS, which is 30,732).

**Table 2:** The effect of living alone on length of stay (2016-2019 and 2020-2023).

		(1)	(2)	(3)	(4)
		Pre-covid	Pre-covid	Post-covid	Post-covid
					
(A)	Live Alone	0.358***	0.232***	0.305***	0.166***
		(0.015)	(0.020)	(0.026)	(0.026)
					
(B)	Live Alone x Public Hospitals		0.184***		0.227***
			(0.027)		(0.045)
					
	Outcome Mean	3.33	3.33	2.67	2.67
	Public Hospital Mean	3.60	3.60	2.98	2.98
	Private Hospital Mean	2.82	2.82	2.19	2.19
	Year FEs	YES	YES	YES	YES
	Casemix Controls	YES	YES	YES	YES
	Hospital FEs	YES	YES	YES	YES
	LSOA FEs	YES	YES	YES	YES
	HRG FEs	YES	YES	YES	YES
	N	158968	158968	60677	60677
(A)+(B)	Live Alone for Public Hospital		0.416***		0.393***
			(0.019)		(0.037)

Notes: This table reports coefficients on the effect of living alone on length of stay before and after the pandemic. Casemix controls include age, sex, ethnicity, pre-operative health, chronic conditions, symptom length, comorbidities, hospital admission experience. Standard errors in parentheses are clustered at the hospital level. * significant at the 10% level, ** significant at the 5% level, *** significant at the 1% level.

In terms of other demographic characteristics, about half of hip replacement patients are over 70 years old, and 40 percent are male. 83 percent of patients are from white ethnic background in the pre-covid period, about 9 percentage points higher than the post-covid period. As for the chronic conditions, arthritis is the most common one (73%), followed by high blood pressure (37%). Differences in these conditions before and after the pandemic is negligible, with the magnitude of one percentage point. Other common chronic conditions include depression, lung disease, diabetes, circulation, cancer, and heart disease (5%-10%).

69% report having hip-related symptoms for between 1 and 5 years prior to the pandemic, which increases to 73% after the pandemic. 19% of patients had symptoms for longer than 5 years, and 20% after the pandemic. This suggests that more patients lived with their hip-related symptoms for a longer period after the pandemic, which is consistent with delayed access to health care following the onset of the pandemic. Patients report on average 1.39 Elixhauser comorbidities, increasing to 1.50 after COVID-19. Over one third of patients have at least one hospital admission in the year prior to surgery, and about 5% have more than three.

As mentioned above, one concern when comparing public and private providers is that they differ in casemix. In Table A4 in the Appendix, we provide the descriptive statistics split for public and private providers before and after COVID-19. They confirm that private providers treat less complex patients. For example, in the pre-covid period, 18% of patients fall in the group with the lowest pre-operative health, while this is the case for only 10% for private providers. The proportion of patients who are older than 80 years old is 15% in public hospitals and 11% in private hospitals. Patients in private hospitals are less likely to have co-morbidities. 61% of patients had no past admissions in public hospitals and 69% for private hospitals. The Elixhauser index is also higher for public hospitals (1.51 vs 1.17).

Table 2 shows the effect of living alone on length of stay before and after the pandemic, presenting the results of estimating equation (1), which pools the effect across all hospitals, and (2), which splits the effect between public and private hospitals. We document the impact of living alone for the pre-COVID-19 and post-COVID-19 periods in columns (1)-(2) and columns (3)-(4), respectively. Column (1) displays the pooled results from eq. (1). It suggests that the length of stay is 0.36 days longer for patients living alone compared to those living with others, which is equivalent to 11% of mean value (3.33 days) and 0.20 standard deviations of length of stay. The coefficient is statistically significant at 1% level after controlling for a range of patient characteristics such as age, sex, pre-surgical health status, and comorbidities, and including time, provider, LSOA, and HRG fixed effects.

Column (2) reports the coefficient from estimating eq. (2). The effect of living alone on length of stay for private hospitals is smaller and equal to 0.23 days. The point estimate for the interaction with public hospitals is positive and statistically significant. It suggests that hip replacement patients who live alone and are treated by public hospitals experience an additional 0.18 days longer stay in hospital than those who live alone but are treated in private hospitals. At the bottom of Column (2), we also present the combined effect of living alone on length of stay in public hospitals, which is the sum of coefficient (A) and coefficient (B). Living alone leads to an increase in length of stay by 0.42 days in public hospitals, which is about double the effect in private hospitals.

Columns (3) and (4) present the corresponding coefficients for the post-COVID-19 period. The estimate in Column (3) shows that living alone increases length of stay by 0.31 days (equivalent to around 11% of mean value), which is similar to that in Column (1) in both sign and magnitude. This suggests that living alone remains an important determinant of length of stay even in the pandemic when a growing shortage of beds was exacerbated and concerns about catching COVID-19 in hospitals was widespread. Column (4) shows consistent findings: patients who live alone stay 0.17 days (6%) longer in private hospitals and 0.39 days (15%) in public hospitals.

In the Appendix, Table A1, we report all the covariates. Older and female patients and patients with worse pre-operative health or higher past hospital admissions have longer length of stay, conditional on other covariates.

Table 3 splits the length of stay between pre-operative and post-operative length of stay. It suggests that the effect of living alone is concentrated on the post-operative length of stay, while there is no effect on pre-operative length of stay, which is much shorter on average. The coefficients of living alone on post-operative length of stay are very similar to those reported in Table 2.

**Table 3:** The effect of living alone on pre-operative and post-operative LOS.

		(1)	(2)	(3)	(4)
		Pre-covid		Post-covid	
		pre-LOS	post-LOS	pre-LOS	post-LOS
					
(A)	Live Alone	0.002	0.232***	0.001	0.165***
		(0.006)	(0.020)	(0.003)	(0.026)
					
(B)	Live Alone x Public Hospitals	0.002	0.180***	-0.003	0.230***
		(0.007)	(0.027)	(0.005)	(0.045)
					
	Outcome Mean	0.05	3.29	0.02	2.64
	Public Hospital Mean	0.02	3.58	0.03	2.95
	Private Hospital Mean	0.09	2.72	0.00	2.19
	Year FEs	YES	YES	YES	YES
	Casemix Controls	YES	YES	YES	YES
	Hospital FEs	YES	YES	YES	YES
	LSOA FEs	YES	YES	YES	YES
	HRG FEs	YES	YES	YES	YES
	N	158968	158968	60677	60677
(A)+(B)	Live Alone for Public Hospital	0.004	0.412***	-0.002	0.395***
		(0.004)	(0.019)	(0.004)	(0.037)

Notes: This table reports coefficients on the effect of living alone on pre-operative and post-operative LOS. Casemix controls include age, sex, ethnicity, pre-operative health, chronic conditions, symptom length, comorbidities, hospital admission experience. Standard errors in parentheses are clustered at the hospital level. * significant at the 10% level, ** significant at the 5% level, *** significant at the 1% level.

Table 4 shows the results from using coarsened exact matching in Columns (2) and (4). To facilitate comparison, we present the baseline results without matching in Columns (1) and (3). Differences in the effect of living alone on length of stay between public and private hospitals could be due to differences in patient casemix. The matching approach allows us to compare length of stay for patients with similar observable characteristics. As expected, the results with the matching procedure shown in Columns (1) and (2) suggest that the interaction term between living alone and public hospitals reduces from 0.18 to 0.14 days. The effect of living alone for public hospitals therefore reduces from 0.42 days to 0.36 days. We find similar results following the pandemic. The effect of living alone on length of stay in public hospitals before and after matching reduces from 0.39 days to 0.32 days. Taken together, these results suggest that differences in patient casemix explain only a small share of the differences in the effect of living alone on length of stay between public and private hospitals, and that the effect is substantially larger in public hospitals even for a given casemix of patients.

**Table 4:** The effect of living alone on length of stay with and without matching.

		(1)	(2)	(3)	(4)
		Pre-covid		Post-covid	
		Without	With	Without	With
		matching	Matching	matching	matching
					
(A)	Live Alone	0.232***	0.223***	0.166***	0.135**
		(0.020)	(0.025)	(0.026)	(0.052)
					
(B)	Live Alone x Public Hospitals	0.184***	0.140***	0.227***	0.189*
		(0.027)	(0.038)	(0.045)	(0.098)
					
	Outcome Mean	3.33	3.00	2.67	2.36
	Public Hospital Mean	3.60	3.23	2.98	2.55
	Private Hospital Mean	2.82	2.78	2.19	2.16
	Year FEs	YES	YES	YES	YES
	Casemix Controls	YES	YES	YES	YES
	Hospital FEs	YES	YES	YES	YES
	LSOA FEs	YES	YES	YES	YES
	HRG FEs	YES	YES	YES	YES
	N	158968	73004	60677	23220
(A)+(B)	Live Alone for Public Hospital	0.416***	0.363***	0.393***	0.324***
		(0.019)	(0.029)	(0.037)	(0.083)

Notes: This table reports coefficients on the effect of living alone on LOS with and without matching. Casemix controls include age, sex, ethnicity, pre-operative health, chronic conditions, symptom length, comorbidities, hospital admission experience. Standard errors in parentheses are clustered at the hospital level. * significant at the 10% level, ** significant at the 5% level, *** significant at the 1% level.

Table 5 investigates whether the inclusion of doctor fixed effects influences our results. Even though we control for supply factors through hospital fixed effects, individual doctors may differ in the way they take clinical and non-clinical factors into account, leading to different discharge decisions and length of stay. We therefore use data on consultant code from HES and include consultant fixed effects in our specification. Over the study period, there are approximately 1,800 hospital specialists in our sample. Columns (2) and (4) report the coefficients from estimating eq. (2) with additional doctor fixed effects for pre-COVID-19 and post-COVID-19 periods, respectively. The results are very similar to those reported in Table 2. Thus, differences in length of stay across doctors within the hospital do not affect our findings.

**Table 5:** The effect of living alone on length of stay with doctor fixed effects.

		(1)	(2)	(3)	(4)
		Pre-covid		Post-covid	
					
(A)	Live Alone	0.232***	0.230***	0.166***	0.160***
		(0.020)	(0.020)	(0.026)	(0.026)
					
(B)	Live Alone x Public Hospitals	0.184***	0.184***	0.227***	0.224***
		(0.027)	(0.028)	(0.045)	(0.046)
					
	Outcome Mean	3.33	3.33	2.67	2.67
	Public Hospital Mean	3.60	3.60	2.98	2.98
	Private Hospital Mean	2.82	2.82	2.19	2.19
	Year FEs	YES	YES	YES	YES
	Casemix Controls	YES	YES	YES	YES
	Hospital FEs	YES	YES	YES	YES
	LSOA FEs	YES	YES	YES	YES
	HRG FEs	YES	YES	YES	YES
	**Doctor FEs**	**NO**	**YES**	**NO**	**YES**
	N	158968	158968	60677	60677
(A)+(B)	Live Alone for Public Hospital	0.416***	0.414***	0.393***	0.384***
		(0.019)	(0.019)	(0.037)	(0.037)

Notes: This table reports coefficients on the effect of living alone on LOS with and without adding doctor fixed effects. Casemix controls include age, sex, ethnicity, pre-operative health, chronic conditions, symptom length, comorbidities, hospital admission experience. Standard errors in parentheses are clustered at the hospital level. * significant at the 10% level, ** significant at the 5% level, *** significant at the 1% level.

Table 6 examines the heterogeneous effects of living alone in the pre-COVID-19 period across several patient characteristics: age, sex, rural/urban, and deprivation. Older people could have a higher demand for follow-up care after surgery and be impacted more strongly by living alone and the lack of support at home. Panel A suggests that this is the case and that the effect of living alone on length of stay is higher among older patients. It reports the corresponding results where the sample is divided into patients above and below the median age (70 years old). As shown in Column (1), patients who live alone and are under 70 years old stay 0.15 days longer (6% of the mean value of 2.64 days) in private hospitals and 0.34 days longer (11% of the mean value of 3.10 days) in public hospitals. For patients aged over 70, the effect of living alone increases to 0.36 days (12% of the mean value of 3.01 days, which is double relative to the effect on patients under 70 years old) for private hospitals. Similarly, the magnitude of the effect in public hospitals increases to 0.64 days, which is equivalent to 16% of the mean value of 4.11 days.

**Table 6:** Heterogeneity analysis. *The effect of living alone on length of stay by age, sex, urban/rural, deprivation, pre-operative health condition and complication (pre-covid sample)*.

		(1)	(2)
**Panel A: Age**		Age<70	Age≥70
			
(A)	Live Alone	0.154***	0.355***
		(0.023)	(0.031)
(B)	Live Alone x Public Hospitals	0.184***	0.281***
		(0.035)	(0.043)
			
	Outcome Mean	2.94	3.75
	Public Hospital Mean	3.10	4.11
	Private Hospital Mean	2.64	3.01
	N	81464	77504
(A)+(B)	Live Alone for Public Hospital	0.337***	0.637***
		(0.026)	(0.030)
**Panel B: sex**		Male	Female
			
(A)	Live Alone	0.254***	0.237***
		(0.036)	(0.019)
(B)	Live Alone x Public Hospitals	0.210***	0.163***
		(0.050)	(0.027)
			
	Outcome Mean	3.10	3.49
	Public Hospital Mean	3.34	3.78
	Private Hospital Mean	2.64	2.93
	N	64256	94712
(A)+(B)	Live Alone for Public Hospital	0.464***	0.400***
		(0.039)	(0.020)
**Panel C: Urban/Rural**		Urban	Rural
			
(A)	Live Alone	0.229***	0.234***
		(0.022)	(0.031)
(B)	Live Alone x Public Hospitals	0.187***	0.181***
		(0.030)	(0.044)
	Outcome Mean	3.39	3.17
	Public Hospital Mean	3.66	3.42
	Private Hospital Mean	2.83	2.78
	N	116548	42386
(A)+(B)	Live Alone for Public Hospital	0.415***	0.415***
		(0.021)	(0.031)
**Panel D: Deprivation**		Less Deprived	More Deprived
			
(A)	Live Alone	0.224***	0.241***
		(0.026)	(0.024)
(B)	Live Alone x Public Hospitals	0.192***	0.175***
		(0.037)	(0.033)
			
	Outcome Mean	3.29	3.38
	Public Hospital Mean	3.56	3.65
	Private Hospital Mean	2.85	2.77
	N	78683	78994
(A)+(B)	Live Alone for Public Hospital	0.416***	0.416***
		(0.026)	(0.023)
**Panel E: Health**		Worse Health	Better Health
			
(A)	Live Alone	0.262***	0.198***
		(0.035)	(0.024)
(B)	Live Alone x Public Hospitals	0.198***	0.162***
		(0.048)	(0.036)
			
	Outcome Mean	3.55	3.12
	Public Hospital Mean	3.82	3.35
	Private Hospital Mean	2.87	2.78
	N	78616	80352
(A)+(B)	Live Alone for Public Hospital	0.460***	0.360***
		(0.031)	(0.027)
**Panel F: Complications**		Less Complicated	More Complicated
			
(A)	Live Alone	0.201***	0.311***
		(0.026)	(0.046)
(B)	Live Alone x Public Hospitals	0.113***	0.191***
		(0.039)	(0.061)
			
	Outcome Mean	2.84	3.69
	Public Hospital Mean	2.98	3.99
	Private Hospital Mean	2.65	2.94
	N	61931	52936
(A)+(B)	Live Alone for Public Hospital	0.314***	0.502***
		(0.028)	(0.041)

Notes: This table reports coefficients on the heterogeneous effect of living alone on length of stay. Casemix controls include age, sex, ethnicity, pre-operative health, chronic conditions, symptom length, comorbidities, hospital admission experience. Less/more deprived patients are defined based on the median indices of multiple deprivation (IMD). Better/worse pre-operative health patients are those with OHS above/below the median value. Standard errors in parentheses are clustered at the hospital level. * significant at the 10% level, ** significant at the 5% level, *** significant at the 1% level.

Panel B presents the heterogeneous effect of living alone on length of stay by sex. We find that the effect is relatively similar for male and female patients for private hospitals (0.25 vs 0.24 days). The effect of living alone is larger for male than female patients (0.46 vs 0.40 days) for public hospitals. Given that the mean length of stay is shorter for males, the differences between males and females is more pronounced in percentage terms: 10% vs 8% longer stays for private hospitals; 14% vs 11% for public hospitals.

Panel C shows the results for urban and rural patients. Rural patients may travel greater distances and this could make arrangements for discharge more complex. The results appear similar across the two groups. Living alone increases length of stay by 0.42 days in public hospitals, which is equivalent to a 11% increase for urban patients and a 12% increase for rural patients.

Panel D, we split patients into more and less deprived groups based on the median value of the Index of Multiple Deprivation (IMD). The estimates are similar to the main results in Table 2: the impact of living alone on length of stay is around 0.42 days for both groups in public hospitals. Nevertheless, the estimated effect is a little larger for more deprived patients in private hospitals than less deprived patients. Specifically, the coefficient is 0.24 days (9%) for the more deprived, while that is 0.22 days (8%) for the less deprived.

Panel E splits the sample between patients who have a value of pre-operative health, as measured by the Oxford Hip Score, which is above or below the median. It shows that the effect of living alone is more pronounced for patients in worse pre-operative health. The impact of living alone on length of stay in private hospitals is around 0.26 days for patients in worse pre-operative health and 0.20 days for those in better pre-operative health. In public hospitals the coefficients are 0.46 and 0.36 days, respectively.

Last, panel F splits the sample between patients with more or less complications. We restrict the sample to patients with “very major hip procedures for non-trauma” (HRG codes HN12A-F), which accounts for 72.3% of the sample. We then divide the sample between patients with a complication score of 0-1 (HRG code HN12F), which accounts for 61,931 patients with an average length of stay of 2.84 days, and patients with complication scores 2 to 10+ (HRG codes HN12A-E), which accounts for 52,936 patients and an average length of stay of 3.69 days. The effect of living alone on length of stay in private hospitals is around 0.20 days for patients with 0-1 complications and 0.31 days for those with more complications. In public hospitals the coefficients are 0.50 and 0.31 days, respectively.^6^

Once ready to be discharged, some patients may want to return to a care home rather than their own home. The search for a suitable care home could explain the delay in discharge and the longer length of stay. However, in our sample very few patients are not discharged to their usual place of residence. About 1% of patients in the pre-covid period and 0.8% in post-covid period are not discharged to their usual place of residence. This could be do a strong preference to return home after surgery or to the limited supply of care homes or rehabilitation facilities. As a robustness check, we conduct our main analysis with a restricted sample where we exclude patients who are not discharged to their usual place of residence. The results are provided in Table 7. The key findings are similar. The effect of living alone on length of stay for public hospitals is 0.40 (vs 0.42 in Table 2) pre-covid, and 0.38 (vs 0.39 in Table 2) post-covid.

**Table 7:** The effect of living arrangement on length of stay, restricted sample excluding patients who are not discharged at home.

		(1)	(2)	(3)	(4)
		Pre-covid	Pre-covid	Post-covid	Post-covid
(A)	Live Alone	0.344***	0.228***	0.291***	0.162***
		(0.015)	(0.014)	(0.026)	(0.026)
(B)	Live Alone x Public Hospitals		0.171***		0.214***
			(0.020)		(0.046)
	Outcome Mean	3.31	3.31	2.65	2.65
	Public Hospital Mean	3.58	3.58	2.96	2.96
	Private Hospital Mean	2.81	2.81	2.19	2.19
	Year FEs	YES	YES	YES	YES
	Casemix Controls	YES	YES	YES	YES
	Hospital FEs	YES	YES	YES	YES
	LSOA FEs	YES	YES	YES	YES
	HRG FEs	YES	YES	YES	YES
	N	157334	157334	60195	60195
(A)+(B)	Live Alone for Public Hospital		0.399***		0.375***
			(0.018)		(0.038)

Notes: This table reports coefficients on the effect of living alone on LOS after excluding patients who are not discharged at home. Casemix controls include age, sex, ethnicity, pre-operative health, chronic conditions, symptom length, comorbidities, hospital admission experience. Standard errors in parentheses are clustered at the hospital level. * significant at the 10% level, ** significant at the 5% level, *** significant at the 1% level.

Our results show that living alone increases length of stay. One possible concern is that our results may reflect people living alone being in worse health. Although we have controlled for a rich set of covariates related to patient health, we cannot completely rule out that unobserved dimensions of health remain. However, we can use our measure of pre-operative health to test whether patients who live alone are more likely to be in worse pre-operative health. In Table 8 we test whether, conditional on other patient covariates and other controls, patients who live alone have worse pre-operative health. We find the opposite. Patients who live alone tend to be in better pre-operative health. Living alone is positively associated with an increase of 0.65 points on the OHS scale (3.8% at the sample mean) in the pre-COVID period and across public and private hospitals. The results are qualitatively similar across the other specifications. This gives indirect support that it is living arrangements that we are capturing, and not unobserved dimensions of health. In Table 9, we further show the results of living alone on length of stay when we omit pre-operative health. Consistently with patients living alone being in better pre-operative health, the effect of living alone on length of stay is smaller when pre-operative health is omitted. However, the difference in coefficients is very small. In column (1), the coefficient is 0.351 days rather than 0.358 in the pre-COVID-19 period (across public and private hospitals) and is 0.301 days in the post-COVID-19 period rather than 0.305.^7^

**Table 8:** The effect of living arrangement on pre-operative health.

		(1)	(2)	(3)	(4)
		Pre-covid	Pre-covid	Post-covid	Post-covid
(A)	Live Alone	0.652***	0.729***	0.373***	0.454**
		(0.061)	(0.096)	(0.127)	(0.181)
					
(B)	Live Alone x Public Hospitals		-0.114		-0.125
			(0.121)		(0.233)
					
	Outcome Mean	17.29	17.29	16.89	16.89
	Public Hospital Mean	16.50	16.50	15.82	15.82
	Private Hospital Mean	18.79	18.79	18.50	18.50
	Year FEs	YES	YES	YES	YES
	Casemix Controls	YES	YES	YES	YES
	Hospital FEs	YES	YES	YES	YES
	LSOA FEs	YES	YES	YES	YES
	HRG FEs	YES	YES	YES	YES
	N	158968	158968	60677	60677
(A)+(B)	Live Alone for Public Hospital		0.615***		0.329**
			(0.077)		(0.164)
					

Notes: This table reports coefficients on the effect of living alone on pre-operative health. Casemix controls include age, sex, ethnicity, pre-operative health, chronic conditions, symptom length, comorbidities, hospital admission experience. Standard errors in parentheses are clustered at the hospital level. * significant at the 10% level, ** significant at the 5% level, *** significant at the 1% level.

**Table 9:** The effect of living arrangement on length of stay 2016-2019 and 2020-2023 without pre-operative health.

		(1)	(2)	(3)	(4)
		Pre-covid	Pre-covid	Post-covid	Post-covid
(A)	Live Alone	0.351***	0.226***	0.301***	0.166***
		(0.015)	(0.020)	(0.026)	(0.026)
					
(B)	Live Alone x Public Hospitals		0.182***		0.219***
			(0.027)		(0.045)
					
	Pre-operative health	NO	NO	NO	NO
	Outcome Mean	3.33	3.33	2.67	2.67
	Public Hospital Mean	3.60	3.60	2.98	2.98
	Private Hospital Mean	2.82	2.82	2.19	2.19
	Year FEs	YES	YES	YES	YES
	Casemix Controls	YES	YES	YES	YES
	Hospital FEs	YES	YES	YES	YES
	LSOA FEs	YES	YES	YES	YES
	HRG FEs	YES	YES	YES	YES
	N	158968	158968	60677	60677
(A)+(B)	Live Alone for Public Hospital		0.408***		0.385***
			(0.019)		(0.037)

Notes: This table reports coefficients on the effect of living alone on length of stay without controlling for pre-operative health. Standard errors in parentheses. * significant at the 10% level, ** significant at the 5% level, *** significant at the 1% level.

A possible explanation for patients who live alone having better pre-operative health is that patients who live with a partner have more support and are more likely to delay the surgery and have the surgery only once health has deteriorated further. Given that we have data on symptom duration, which we also use as a control variable, we can check whether people who live alone are less likely to tolerate hip problems leading to a longer symptom duration. We find that the distribution of symptom duration is very similar for those living alone and those not living alone (see Table A5 in the Appendix). More precisely, the proportion of patients in the pre-covid period who have symptoms between one and five years is 69-70%, between six and ten years is 11-12%, and over ten years is 6-7%. The distribution is similar also in the post-covid period. We have also re-run our analysis omitting symptom duration as a control variable, and the results are virtually the same. (see Table A6 in the Appendix).

Our analysis controls for pre-operative health that relates specifically to the hip. Other dimensions of general health could be correlated both with living alone and length of stay. Through the PROMs data, we have access to EQ-5D data, which is a standard measure of health status, both pre- and post-surgery. These measures are highly correlated with the measures of health captured by the Oxford Hip Score. Moreover, we already control for the type of comorbidities, such as if the patient had diabetes or heart conditions. As a robustness check, we test whether further controlling for pre-operative health, as measured by the EQ-5D, affects the results. Table A2 in the Appendix shows that the results of living alone on length of stay remain very similar. Similarly, in Table A3, we show that living alone has a small positive association with pre-operative health as measured by the EQ-5D in the pre-COVID-19 period, and no association in the post-COVID-19 period.

The analysis has focused on the effect of living alone on length of stay. Lack of support at home could also affect rehabilitation post-surgery leading to worse health outcomes. Table 10 shows the association between living alone and post-operative health status measured by the Oxford Hip Score before and after the pandemic. Column (1) shows that prior to the pandemic, living alone reduces post-operative health by 0.26 points on the OHS scale. The effect appears quantitatively small relative to the mean value of 40.25 (less than 1%). In column (2), we do not find a statistically significant estimate for patients in private hospitals, while the effect for public hospitals is negative and significant but remains small in magnitude. In the post-COVID-19 period, the estimates are small and statistically insignificant for both public and private hospitals.

**Table 10:** The effect of living arrangement on post-operative health.

		(1)	(2)	(3)	(4)
		Pre-covid	Pre-covid	Post-covid	Post-covid
					
(A)	Live Alone	-0.257***	-0.124	-0.147	0.327
		(0.070)	(0.110)	(0.227)	(0.327)
					
(B)	Live Alone x Public Hospitals		-0.190		-0.708
			(0.144)		(0.450)
					
	Outcome Mean	40.25	40.25	40.09	40.09
	Public Hospital Mean	39.51	39.51	39.51	39.51
	Private Hospital Mean	41.64	41.64	40.98	40.98
	Year FEs	YES	YES	YES	YES
	Casemix Controls	YES	YES	YES	YES
	Hospital FEs	YES	YES	YES	YES
	LSOA Fes	YES	YES	YES	YES
	HRG Fes	YES	YES	YES	YES
	N	122979	122979	30732	30732
(A)+(B)	Live Alone for Public Hospital		-0.314***		-0.381
			(0.091)		(0.302)

Notes: This table reports coefficients on the effect of living alone on post-operative health. Casemix controls include age, sex, ethnicity, pre-operative health, chronic conditions, symptom length, comorbidities, hospital admission experience. Standard errors in parentheses are clustered at the hospital level. * significant at the 10% level, ** significant at the 5% level, *** significant at the 1% level.

## Discussion

We have investigated the effect of living alone on hospital length of stay. On one hand, patients who live alone may lack the support once discharged home to take care of themselves, and this may delay the hospital’s discharge even if the patient is clinically fit. On the other hand, individuals who live alone can draw on family and friends to help them once discharged. Hip replacement is a common planned procedure. Therefore, patients have time to plan for the surgery and to prepare some informal or paid support (e.g. someone to take care of the meals, cleaning the house, etc.) once returning home. Rehabilitation from hip replacement also follows standard protocols that can be explained in advance. Our results suggest that the first effect dominates and patients who live alone are not able to be discharged as quickly as those who live with other people (e.g. a spouse), and that providers allow patients to stay longer when patients expect difficulties with the discharge.

The effect also appears to be substantive. Across public and private hospitals, patients who live alone stay about 11% longer in the pre-pandemic period. The findings are consistent with previous qualitative studies from different contexts and countries. A previous study suggests that nurses in Norway feel responsible towards old patients who live alone in rural areas (Ness et al., 2015). One small-scale study on coronary bypass in Finland found that patients who live alone were more likely to experience depressive symptoms before surgery (Okkonen and Vanhanen, 2006), which could induce doctors to keep patients longer. However, in our study we have shown that patients who live alone are generally in better pre-operative health. Another study from the UK found that patients with cancer who live alone perceived emotional and practical barriers to accessing care (Hanratty et al., 2013). A study from the US suggested that providers found more challenging to support patients with cognitive impairments who live alone (Portacolone et al., 2023).

Many OECD countries rely on provision by private providers in addition to public providers to treat publicly-funded patients (Siciliani et al., 2022). In England, the proportion of patients treated by private providers has increased over time with more than a third of hip-replacement patients being treated privately. This gives an opportunity to test whether the effect of living alone differs between public and private providers. A stronger concern towards profits may induce private providers to contain costs (Glaeser and Shleifer, 2001; Brekke et al., 2012). In the context of the health sector, the profit motive can reduce length of stay and minimise the impact of non-clinical considerations such as living alone therefore implementing discharge protocols more rigidly. Our analysis is in line with this behaviour and suggests that the effect of living alone is smaller for private hospitals both in absolute and relative terms. The effect of living alone on length of stay is 0.23 days or 8% for private hospitals and 0.42 days or 12% for public hospitals. A typical concern with provision by private providers is that they have a stronger incentive to treat patients with lower complexity leading to a lighter casemix. Our matching analysis shows that the effect of living alone for public providers reduces only to a small extent once patients in public hospitals are matched with patients with similar patient characteristics in private hospitals. The effect of living alone on length of stay reduces to 0.36 days in public hospitals. But given that the mean length of stay is also smaller and equal to three days in public hospitals, the effect of living alone is 12% for public hospitals after matching. (The effect for private hospitals remains the same given that nearly all patients treated by private hospitals are matched with one or more patients treated by public hospitals). This suggests again that it is mostly the concern towards increasing profits that affects the impact of living alone on length of stay in private providers.

One possible mechanism through which living alone increases length of stay is through delays in obtaining a place in a care home or a rehabilitation centre. Although some patients may plan ahead, others may realise only after the surgery that they need help and seek a place in a care home or rehabilitation centre. In our analysis, we can rule out that this mechanism is at work. This is because only very few patients do not return home following discharge. The results are very similar once these patients are excluded from the sample.

Driven by technological innovation, length of stay has reduced steadily over our sample period, and this includes the post-pandemic period, which could attenuate the effect of living alone. Following COVID-19, the effect of living alone on length of stay is smaller when measured in days for both public and private providers but is similar in percentage terms (and equal to 11%). The effect is however somewhat smaller for private hospitals (6%) and larger in public hospitals (15%). We therefore conclude that the effect of living alone does not appear to have changed qualitatively before and after COVID-19.

Policies aimed at reducing length of stay could target patients where the effect of living alone is quantitatively more important. We have investigated the heterogenous effect of living alone across a range of patient characteristics. We find that living alone has a much larger impact on older patients. The effect is about double for patients who are over 70 years old relative to those who are less than 70 years old. This suggests that older patients could be targeted as potentially needing more support to be discharged promptly. This could involve contacting patients in advance and ensuring that patients have planned for adequate support following discharge. We instead do not find significant differences in other domains. The effect is only a little larger for men, while it is similar between patients in urban and rural areas, and also patients living differing in socioeconomic status, which we measure through income deprivation.

Our findings can be used to conduct back-of-the-envelope computations of interventions that facilitate earlier discharge for those living alone. Suppose for example that the effect of living alone on length of stay in public hospitals could be reduced by 0.2 days, about half of the effect. Given an average annual volume of hip replacements of around 96,000 over our sample period, and 25% of those patients living alone, this reduction would translate into freeing up 4,800 beddays (96,000*0.2*0.25). Given an average length of stay of 3.33 days, this is equivalent to 1.52% of the total beddays required for hip replacement in a given year (4,800/(3.33*96,000)) or 13.15 additional beds (4,800/365). Such additional capacity can be used to treat more patients from the waiting list. For example, 1,200 additional patients could receive surgeries with an average length of stay of four days or 1,600 patients with an average length of stay of three days. This suggests that there is further scope for policies that support patients who live alone to facilitate a quicker discharge from the hospital.

In terms of limitations, although we cannot rule out that unobserved dimensions of health remain, our analysis controls for a range of detailed patient characteristics, including a condition-specific assessment of pre-operative health, past admissions. Even adding a measure of general health does not change our results. On the supply side, the analysis controls for hospital fixed effects and is robust to the inclusion of doctor fixed effects that control for variation in length of stay at the specialist level. We are only able to observe if patients live alone and we do not have access to more detailed information about the contacts that patients have with other members of the community. We however control for small area (LSOA) effects, which control for local supply in the availability of care homes and rehabilitation services. These could potentially control for some differences in informal networks within communities that vary across small areas comprising on average 1,500 inhabitants. Similarly, we cannot differentiate whether the patient lives with a spouse, partner, family or friends.

## Conclusion

Our study highlights that non-clinical factors and personal circumstances, such as living arrangements, can affect provider behaviour and the resources used by healthcare providers for elderly patients. This is the case for both public and private providers treating publicly-funded patients. The findings imply that there is scope for policies that better support the discharge of patients and the return to their own home.

## CRediT authorship contribution statement

**Luigi Siciliani:** Writing – original draft, Visualization, Validation, Supervision, Software, Resources, Methodology, Investigation, Funding acquisition, Formal analysis, Data curation, Conceptualization. **Jinglin Wen:** Writing – original draft, Validation, Software, Resources, Project administration, Methodology, Investigation, Formal analysis, Data curation, Conceptualization. **James Gaughan:** Writing – review & editing, Supervision, Software, Resources, Methodology, Investigation, Formal analysis, Conceptualization.

## Figures and Tables

**Table A1:** The effect of living arrangement on length of stay (2016-2019; 2020-2023).

	(1)	(2)	(3)	(4)
	Pre-covid	Pre-covid	Post-covid	Post-covid
Live Alone	0.358***	0.232***	0.305***	0.166***
	(0.015)	(0.020)	(0.026)	(0.026)
Live Alone x Public Hospitals		0.184***		0.227***
		(0.027)		(0.045)
Public Hospitals		0.773***		0.262
		(0.109)		(0.229)
Under 30	-0.054	0.173	0.044	-0.142
	(0.092)	(0.194)	(0.316)	(0.225)
[30, 60)	-0.339***	-0.139***	-0.266***	-0.137***
	(0.017)	(0.016)	(0.029)	(0.029)
[60, 65)	-0.271***	-0.121***	-0.204***	-0.086***
	(0.017)	(0.017)	(0.031)	(0.032)
[65, 70)	-0.175***	-0.093***	-0.127***	-0.053*
	(0.014)	(0.015)	(0.030)	(0.030)
[75, 80)	0.260***	0.157***	0.169***	0.112***
	(0.017)	(0.020)	(0.033)	(0.036)
Over 80	0.838***	0.490***	0.712***	0.391***
	(0.026)	(0.027)	(0.055)	(0.045)
Under 30 x Public Hospitals		-0.291		0.198
		(0.217)		(0.426)
[30, 60) x Public Hospitals		-0.308***		-0.229***
		(0.026)		(0.054)
[60, 65) x Public Hospitals		-0.249***		-0.218***
		(0.026)		(0.053)
[65, 70) x Public Hospitals		-0.136***		-0.134***
		(0.024)		(0.051)
[75, 80) x Public Hospitals		0.160***		0.101
		(0.030)		(0.062)
Over 80 x Public Hospitals		0.486***		0.471***
		(0.038)		(0.085)
Male	-0.278***	-0.217***	-0.314***	-0.209***
	(0.013)	(0.017)	(0.030)	(0.024)
Male x Public Hospitals		-0.100***		-0.183***
		(0.023)		(0.047)
Multiple ethnicities	0.245*	0.206	0.251	0.062
	(0.138)	(0.174)	(0.360)	(0.225)
Asian ethnicity	0.350***	0.125	0.319*	0.361*
	(0.072)	(0.111)	(0.193)	(0.198)
African ethnicity	0.263***	-0.030	0.216	0.087
	(0.079)	(0.130)	(0.152)	(0.227)
Other ethnicity	0.127	-0.140	-0.029	0.117
	(0.105)	(0.207)	(0.145)	(0.312)
Unknown ethnicity	-0.039**	-0.018	-0.013	0.004
	(0.016)	(0.014)	(0.022)	(0.024)
Multiple ethnicities x Public Hospitals		0.053		0.217
		(0.239)		(0.546)
Asian ethnicity x Public Hospitals		0.320**		0.004
		(0.146)		(0.281)
African ethnicity x Public Hospitals		0.372**		0.159
		(0.168)		(0.305)
Other ethnicity x Public Hospitals		0.321		-0.148
		(0.229)		(0.337)
Unknown ethnicity x Public Hospitals		-0.015		0.002
		(0.029)		(0.047)
preop_0to8	0.387***	0.153***	0.388***	0.144***
	(0.024)	(0.024)	(0.040)	(0.035)
preop_9to16	0.151***	0.082***	0.149***	0.055**
	(0.014)	(0.014)	(0.026)	(0.025)
preop_17to24	0.069***	0.028**	0.098***	0.056*
	(0.013)	(0.013)	(0.027)	(0.029)
preop_33plus	-0.057**	-0.058**	0.016	0.043
	(0.025)	(0.023)	(0.042)	(0.049)
preop_0to8 x Public Hospitals		0.313***		0.367***
		(0.036)		(0.067)
preop_9to16 x Public Hospitals		0.114***		0.177***
		(0.022)		(0.047)
preop_17to24 x Public Hospitals		0.073***		0.104**
		(0.022)		(0.052)
preop_33plus x Public Hospitals		-0.007		-0.043
		(0.046)		(0.080)
HEART_DISEASE	-0.047**	-0.094***	-0.239***	-0.325***
	(0.023)	(0.030)	(0.048)	(0.051)
HIGH_BP	-0.106***	-0.013	-0.102***	-0.044*
	(0.012)	(0.014)	(0.025)	(0.024)
STROKE	0.369***	0.029	0.187**	0.214*
	(0.052)	(0.066)	(0.091)	(0.128)
CIRCULATION	0.299***	0.153***	0.235***	0.055
	(0.029)	(0.049)	(0.065)	(0.068)
LUNG_DISEASE	0.099***	0.117***	0.158***	0.089**
	(0.018)	(0.021)	(0.033)	(0.045)
DIABETES	-0.045**	-0.086***	0.011	-0.007
	(0.022)	(0.025)	(0.038)	(0.042)
KIDNEY_DISEASE	0.228***	0.128**	0.210***	0.042
	(0.042)	(0.058)	(0.076)	(0.074)
NERVOUS_SYSTEM	0.428***	0.262***	0.572***	0.291***
	(0.059)	(0.074)	(0.131)	(0.110)
LIVER_DISEASE	-0.086	-0.057	-0.104	-0.244
	(0.071)	(0.086)	(0.135)	(0.162)
CANCER	0.001	0.022	-0.044	-0.099**
	(0.023)	(0.025)	(0.045)	(0.048)
DEPRESSION	0.154***	0.128***	0.159***	0.206***
	(0.017)	(0.022)	(0.032)	(0.040)
ARTHRITIS	-0.006	-0.000	-0.026	0.014
	(0.010)	(0.012)	(0.023)	(0.025)
HEART_DISEASE x Public Hospitals		0.040		0.137
		(0.037)		(0.083)
HIGH_BP x Public Hospitals		-0.120***		-0.078*
		(0.022)		(0.041)
STROKE x Public Hospitals		0.410***		-0.061
		(0.091)		(0.166)
CIRCULATION x Public Hospitals		0.157***		0.228**
		(0.059)		(0.112)
LUNG_DISEASE x Public Hospitals		-0.029		0.113*
		(0.031)		(0.060)
DIABETES x Public Hospitals		0.070*		0.039
		(0.036)		(0.067)
KIDNEY_DISEASE x Public Hospitals		0.102		0.190
		(0.077)		(0.120)
NERVOUS_SYSTEM x Public Hospitals		0.245**		0.490**
		(0.111)		(0.238)
LIVER_DISEASE x Public Hospitals		-0.045		0.193
		(0.123)		(0.258)
CANCER x Public Hospitals		-0.027		0.089
		(0.038)		(0.081)
DEPRESSION x Public Hospitals		0.049		-0.060
		(0.031)		(0.058)
ARTHRITIS x Public Hospitals		-0.015		-0.068
		(0.018)		(0.045)
Symptom length < 1 year	-0.044***	-0.033**	0.025	0.041
	(0.015)	(0.013)	(0.037)	(0.036)
Symptom length 6to10 years	0.040***	0.011	0.030	0.079***
	(0.014)	(0.016)	(0.030)	(0.026)
Symptom length > 10 years	0.094***	0.008	0.094***	0.022
	(0.018)	(0.023)	(0.036)	(0.042)
Count of elixhauser comorbidities	0.034***	-0.066***	-0.067***	-0.126***
	(0.008)	(0.009)	(0.015)	(0.015)
One admission in last year	0.005	-0.010	0.003	-0.012
	(0.011)	(0.013)	(0.025)	(0.026)
Two admissions in last year	0.051**	0.017	-0.014	-0.003
	(0.020)	(0.022)	(0.044)	(0.055)
Three admissions in last year	0.046	-0.006	0.044	-0.074
	(0.036)	(0.046)	(0.077)	(0.071)
Over three admissions in last year	0.152***	0.031	-0.113	-0.067
	(0.043)	(0.061)	(0.093)	(0.104)
One emergency admission in last year	0.293***	0.110***	0.323***	0.132***
	(0.029)	(0.036)	(0.046)	(0.049)
Two emergency admissions in last year	0.569***	0.214**	0.575***	0.345**
	(0.062)	(0.099)	(0.136)	(0.140)
Over two emergency admissions in last year	1.201***	0.340	1.614***	1.144
	(0.248)	(0.357)	(0.479)	(1.359)
Symptom length < 1 year x Public Hospitals		-0.004		-0.024
		(0.026)		(0.070)
Symptom length 6to10 years x Public Hospitals		0.044*		-0.092*
		(0.025)		(0.049)
Symptom length > 10 years x Public Hospitals		0.138***		0.121**
		(0.031)		(0.061)
Count of elixhauser comorbidities x Public Hospitals		0.135***		0.088***
		(0.013)		(0.022)
One admission in last year x Public Hospitals		0.025		0.025
		(0.020)		(0.047)
Two admissions in last year x Public Hospitals		0.052		-0.024
		(0.035)		(0.083)
Three admissions in last year x Public Hospitals		0.070		0.164
		(0.065)		(0.131)
Over three admissions in last year x Public Hospitals		0.148*		-0.081
		(0.081)		(0.161)
One emergency admission in last year x Public Hospitals		0.230***		0.253***
		(0.050)		(0.079)
Two emergency admissions in last year x Public Hospitals		0.404***		0.296
		(0.127)		(0.231)
Over two emergency admissions in last year x Public Hospitals		0.891**		0.582
		(0.443)		(1.371)
Year 2017	-0.143***	-0.073***		
	(0.025)	(0.027)		
Year 2018	-0.346***	-0.200***		
	(0.034)	(0.039)		
Year 2019	-0.547***	-0.366***		
	(0.046)	(0.045)		
Year 2021			-0.104***	-0.216***
			(0.040)	(0.044)
Year 2022			-0.197***	-0.330***
			(0.036)	(0.041)
Year 2023			-0.379***	-0.590***
			(0.082)	(0.095)
2017 x Public Hospitals		-0.109***		
		(0.037)		
2018 x Public Hospitals		-0.225***		
		(0.050)		
2019 x Public Hospitals		-0.277***		
		(0.071)		
2021 x Public Hospitals				0.164**
				(0.072)
2022 x Public Hospitals				0.210***
				(0.067)
2023 x Public Hospitals				0.359**
				(0.151)
N	158968	158968	60677	60677

Notes: This table reports coefficients on the effect of living alone on LOS (full table). Casemix controls include age, sex, ethnicity, pre-operative health, chronic conditions, symptom length, comorbidities, hospital admission experience. Standard errors in parentheses are clustered at the hospital level. * significant at the 10% level, ** significant at the 5% level, *** significant at the 1% level.

**Table A2:** The effect of living arrangement on length of stay with and without ED5Q control.

		(1)	(2)	(3)	(4)
		Pre-covid		Post-covid	
(A)	Live Alone	0.232***	0.226***	0.166***	0.171***
		(0.020)	(0.020)	(0.026)	(0.026)
(B)	Live Alone x Public Hospitals	0.184***	0.187***	0.227***	0.210***
		(0.027)	(0.028)	(0.045)	(0.045)
	EQ5D Index		-0.183***		-0.182***
			(0.023)		(0.043)
	Outcome Mean	3.33	3.32	2.67	2.66
	Public Hospital Mean	3.60	3.59	2.98	2.97
	Private Hospital Mean	2.82	2.81	2.19	2.19
	Year FEs	YES	YES	YES	YES
	Casemix Controls	YES	YES	YES	YES
	Hospital FEs	YES	YES	YES	YES
	LSOA FEs	YES	YES	YES	YES
	HRG FEs	YES	YES	YES	YES
	**EQ5D Control**	**NO**	**YES**	**NO**	**YES**
	N	158968	150655	60677	57851
(A)+(B)	Live Alone for Public Hospital	0.416***	0.413***	0.393***	0.382***
		(0.019)	(0.020)	(0.037)	(0.036)

Notes: This table reports coefficients on the effect of living alone on LOS with and without controlling EQ5D. EQ5D index is a variable that ranges from 0 to 1, with 1 representing full health and 0 representing a state of being dead. Casemix controls include age, sex, ethnicity, chronic conditions, symptom length, comorbidities, hospital admission experience. Standard errors in parentheses are clustered at the hospital level. * significant at the 10% level, ** significant at the 5% level, *** significant at the 1% level.

**Table A3:** The effect of living arrangement on general health measured by EQ5D.

		(1)	(2)	(3)	(4)
		Pre-covid	Pre-covid	Post-covid	Post-covid
(A)	Live Alone	0.005**	0.003	0.001	0.002
		(0.002)	(0.004)	(0.005)	(0.008)
(B)	Live Alone x Public Hospitals		0.003		-0.002
			(0.005)		(0.010)
	Outcome Mean	0.33	0.33	0.31	0.31
	Public Hospital Mean	0.30	0.30	0.27	0.27
	Private Hospital Mean	0.39	0.39	0.37	0.37
	Year FEs	YES	YES	YES	YES
	Casemix Controls	YES	YES	YES	YES
	Hospital FEs	YES	YES	YES	YES
	LSOA FEs	YES	YES	YES	YES
	HRG FEs	YES	YES	YES	YES
	N	150655	150655	57851	57851
(A)+(B)	Live Alone for Public Hospital		0.007**		-0.000
			(0.003)		(0.007)

Notes: This table reports coefficients on the effect of living alone on pre-operative health measured by EQ5D. Casemix controls include age, sex, ethnicity, chronic conditions, symptom length, comorbidities, hospital admission experience. Standard errors in parentheses are clustered at the hospital level. * significant at the 10% level, ** significant at the 5% level, *** significant at the 1% level.

**Table A4:** Summary statistics by public and private hospitals.

	*2016-2019*		*2020-2023*	
	Public	Private	Public	Private
Length of Stay	3.60	2.82	2.98	2.19
Pre-operative	0.02	0.09	0.03	0.00
Post-operative	3.58	2.72	2.95	2.19
Pre-operative Oxford Hip Score (OHS)				
0 to 8	0.18	0.10	0.21	0.11
9 to 16	0.36	0.32	0.36	0.32
17 to 24	0.30	0.34	0.27	0.33
25 to 32	0.14	0.19	0.12	0.20
Over 33	0.04	0.05	0.03	0.05
Post-operative OHS	39.51	41.64	39.51	40.98
Live with Spouse	0.74	0.78	0.74	0.77
Live Alone	0.26	0.22	0.26	0.23
Age Band				
Under 30	0.01	0.00	0.01	0.00
[30, 60)	0.21	0.20	0.22	0.21
[60, 65)	0.12	0.14	0.13	0.15
[65, 70)	0.16	0.19	0.15	0.17
[70, 75)	0.19	0.21	0.19	0.20
[75, 80)	0.16	0.15	0.16	0.16
Over 80	0.15	0.11	0.15	0.11
Male	0.41	0.40	0.40	0.4
White	0.87	0.74	0.84	0.59
Other Ethnicity	0.00	0.00	0.01	0.00
Unknown Ethnicity	0.11	0.25	0.13	0.40
Chronic Conditions				
Heart Disease	0.10	0.05	0.09	0.05
High Blood Pressure	0.38	0.35	0.36	0.35
Stroke	0.02	0.01	0.02	0.01
Circulation	0.06	0.03	0.05	0.03
Lung Disease	0.10	0.06	0.10	0.07
Diabetes	0.10	0.07	0.10	0.07
Kidney Disease	0.02	0.01	0.03	0.01
Nervous Symptom	0.01	0.01	0.01	0.01
Liver Disease	0.01	0.00	0.01	0.01
Cancer	0.06	0.05	0.06	0.05
Depression	0.11	0.08	0.12	0.09
Arthritis	0.72	0.74	0.70	0.75
Symptom Length				
Under 1 Year	0.10	0.14	0.06	0.07
1 to 5 Years	0.70	0.69	0.73	0.74
6 to 10 Years	0.12	0.11	0.14	0.13
Over 10 Years	0.07	0.06	0.08	0.06
Elixhauser Index	1.51	1.17	1.65	1.29
Past Admission Last Year				
No Admission	0.61	0.69	0.66	0.72
One Admission	0.25	0.22	0.22	0.20
Two Admissions	0.08	0.06	0.07	0.05
Three Admissions	0.03	0.02	0.03	0.02
Over Three Admissions	0.02	0.02	0.02	0.01
Past Emergency Admission Last Year				
No Admission	0.91	0.95	0.90	0.94
One Admission	0.07	0.04	0.08	0.05
Two Admissions	0.01	0.01	0.01	0.01
Over Two Admissions	0.00	0.00	0.00	0.00

Note: This table presents the mean values for each variable. The sample size for variables before Covid is 104,557 for public hospitals and 54,411 for private hospitals (except post oxford score, which is 80,277 for public hospitals and 42,702 for the private hospital), while the sample size after Covid is 36,444 for public hospitals and 24,233 for private hospitals (except post-operative health, which are 18,495 for the public hospitals and 12,237 for the private hospitals).

**Table A5:** The distribution of symptom duration.

	Pre-covid	Pre-covid	Post-covid	Post-covid
Symptom duration	Living alone	Not living alone	Living alone	Not living alone
Under 1 Year	0.13	0.11	0.07	0.06
1 to 5 Years	0.70	0.69	0.74	0.73
6 to 10 Years	0.11	0.12	0.13	0.14
Over 10 Years	0.06	0.07	0.06	0.07

Note: This table reports the distribution of symptom duration by patients who live alone and live with spouse.

**Table A6:** The effect of living arrangement on length of stay without controlling for symptom duration.

		(1)	(2)	(3)	(4)
		Pre-covid	Pre-covid	Post-covid	Post-covid
					
(A)	Live Alone	0.358***	0.232***	0.306***	0.166***
		(0.015)	(0.020)	(0.026)	(0.026)
(B)	Live Alone x Public Hospitals		0.184***		0.227***
			(0.027)		(0.045)
	Outcome Mean	3.33	3.33	2.67	2.67
	Public Hospital Mean	3.60	3.60	2.98	2.98
	Private Hospital Mean	2.82	2.82	2.19	2.19
	Year FEs	YES	YES	YES	YES
	**Symptom Duration Control**	**NO**	**NO**	**NO**	**NO**
	Other Casemix Controls	YES	YES	YES	YES
	Hospital FEs	YES	YES	YES	YES
	LSOA FEs	YES	YES	YES	YES
	HRG FEs	YES	YES	YES	YES
	N	158968	158968	60677	60677
(A)+(B)	Live Alone for Public Hospital		0.416***		0.393***
			(0.019)		(0.037)

Notes: This table reports coefficients on the effect of living alone on length of stay before and after the pandemic without controlling for symptom length. Standard errors in parentheses. * significant at the 10% level, ** significant at the 5% level, *** significant at the 1% level.

**Table A7:** The effect of living arrangement on length of stay by pre-covid years.

		(1)	(2)	(3)	(4)	(5)	(6)	(7)	(8)
		2016/17		2017/18		2018/19		2019/20	
(A)	Live Alone	0.401***	0.177***	0.379***	0.246***	0.342***	0.287***	0.339***	0.208***
		(0.064)	(0.058)	(0.038)	(0.051)	(0.033)	(0.041)	(0.034)	(0.040)
(B)	Live Alone x Public Hospitals		0.337***		0.183***		0.074		0.201***
			(0.092)		(0.069)		(0.067)		(0.062)
	Outcome Mean	3.63	3.63	3.47	3.47	3.25	3.25	3.06	3.06
	Public Hospital Mean	3.92	3.92	3.72	3.72	3.51	3.51	3.31	3.31
	Private Hospital Mean	3.01	3.01	2.89	2.89	2.79	2.79	2.65	2.65
	Casemix Controls	YES	YES	YES	YES	YES	YES	YES	YES
	Hospital FEs	YES	YES	YES	YES	YES	YES	YES	YES
	LSOA FEs	YES	YES	YES	YES	YES	YES	YES	YES
	HRG FEs	YES	YES	YES	YES	YES	YES	YES	YES
	N	32571	32571	41364	41364	41773	41773	43260	43260
(A)+(B)	Live Alone for Public Hospital		0.514***		0.429***		0.361***		0.409***
			(0.080)		(0.049)		(0.048)		(0.046)

Notes: This table reports coefficients on the effect of living alone on length of stay by pre-covid year. Standard errors in parentheses. * significant at the 10% level, ** significant at the 5% level, *** significant at the 1% level.

**Table A8:** The effect of living arrangement on survey assistance.

	(1)	(2)	(3)	(4)
	Pre-covid	Pre-covid	Post-covid	Post-covid
Pre-operative Oxford Hip Score (OHS)				
0 to 8	0.176***	0.162***	0.130***	0.120***
	(0.005)	(0.006)	(0.011)	(0.011)
				
9 to 16	0.073***	0.068***	0.046***	0.040***
	(0.003)	(0.003)	(0.008)	(0.008)
				
17 to 24	0.028***	0.026***	0.017***	0.013*
	(0.003)	(0.003)	(0.006)	(0.007)
Over 33	-0.012***	-0.011**	-0.016	-0.019*
	(0.004)	(0.005)	(0.010)	(0.010)
Pre-operative OHS x Living alone				
0 to 8 x Living alone		0.037***		0.024*
		(0.008)		(0.013)
9 to 16 x Living alone		0.003		0.008
		(0.004)		(0.008)
				
17 to 24 x Living alone		-0.007		0.001
		(0.004)		(0.009)
25 to 32 x Living alone		-0.017***		-0.018*
		(0.006)		(0.010)
Over 33 x Living alone		-0.019*		-0.002
		(0.010)		(0.020)
Outcome Mean	0.16	0.16	0.14	0.14
Year FEs	YES	YES	YES	YES
Casemix Controls	YES	YES	YES	YES
Hospital FEs	YES	YES	YES	YES
LSOA FEs	YES	YES	YES	YES
HRG FEs	YES	YES	YES	YES
N	157978	157978	60180	60180

Notes: This table reports coefficients on the effect of pre-operative health on survey assistance before and after the pandemic. The dependent variable is an indicator of whether a patient was assisted to complete the survey. Standard errors in parentheses. * significant at the 10% level, ** significant at the 5% level, *** significant at the 1% level.

**Table A9:** The effect of living arrangement on length of stay (pre-operative health score 9-24).

		(1)	(2)	(3)	(4)
		Pre-covid	Pre-covid	Post-covid	Post-covid
(A)	Live Alone	0.337***	0.227***	0.307***	0.172***
		(0.018)	(0.022)	(0.032)	(0.032)
(B)	Live Alone x Public Hospitals		0.163***		0.216***
			(0.032)		(0.057)
	Outcome Mean	3.28	3.28	2.61	2.61
	Public Hospital Mean	3.53	3.53	2.90	2.90
	Private Hospital Mean	2.82	2.82	2.19	2.19
	Year FEs	YES	YES	YES	YES
	Casemix Controls	YES	YES	YES	YES
	Hospital FEs	YES	YES	YES	YES
	LSOA FEs	YES	YES	YES	YES
	HRG FEs	YES	YES	YES	YES
	N	104059	104059	38839	38839
(A)+(B)	Live Alone for Public Hospital		0.389***		0.388***
			(0.024)		(0.048)

Notes: This table reports coefficients on the effect of living alone on length of stay before and after the pandemic when restricting sample into patients with pre-operative oxford health score between 9 and 24. Standard errors in parentheses. * significant at the 10% level, ** significant at the 5% level, *** significant at the 1% level.

## Appendix

### Theoretical framework

Totally differentiating the equilibrium length of stay with respect to the weight given to profits, we obtain $\frac{\;\partial l^{i*}}{\;\partial \delta^{i}}=\frac{c_{l}}{V_{ll}^{i}}<0.$ A higher weight on profits reduces length of stay. Similarly, we obtain: $\frac{\;\partial l^{i*}}{\;\partial a}\;=-\frac{\alpha^{i}\;\left(h_{la}+\gamma^{i}\;u_{la}\right)}{V_{ll}^{i}}$. This suggests that length of stay is longer when living alone increases the health and utility gain from a marginal increase in length of stay. Although living alone is a dichotomous variable, by continuity, the effect is qualitatively similar by differentiating with respect to *a*.

We can also test how the effect of living alone on length of stay differs between public and private providers by looking at the cross-partial derivative: $\frac{\;\partial l^{i*}}{\;\partial a\partial \delta^{i}}\;=-\frac{\alpha^{i}\;\left(h_{la}+\gamma^{i}\;u_{la}\right)c_{ll}+\;\alpha^{i}\;\left(h_{lla}+\gamma^{i}\;u_{lla}\right)c_{l}}{{\left(V_{ll}^{i}\right)}^{2}}.$ The first effect is negative. Providers that give a higher weight to profits have a higher marginal cost and a more convex cost function, which dampens the effect of living alone on length of stay. The second term is indeterminate and depends on whether the degree of concavity of the health and utility function with respect to length of stay increases or reduces for patients living alone.

## Footnotes

1 The linkage outcome is consistent with the official statistics of linkage rate from the NHS Digital (2024).

2 The inclusion of patients answering living in a nursing home, hospital or other long-term care home or other location does not affect our results.

3 There are many missing values for the post-operative Oxford Hip Score variable in 2022/23, as only 13.5% of all post-operative questionnaires issued were returned (NHS Digital, 2024).

4 We define the pre-Covid-19 period and post-Covid-19 period based on the admission year. If patients are admitted before 2020, they are categorised into the pre-covid period, and vice versa.

5 Stata code cem is used to perform the matching estimation.

6 In Table A7, we also provide heterogeneity analysis by year in the pre-covid period when the sample size for each year is higher. The effect appears relatively stable across years. Average length of stay reduced gradually over time, from 3.63 days in 2016/17 to 2.06 days in 2019/20. The effect of living alone on length of stay also reduced gradually from 0.40 days to 0.34 days. The results do not appear qualitatively different for 2019/20 when the penalties for readmissions within 30 days were eliminated for both public and private providers.

7 A different concern is that patients in worst pre-operative health may be less likely to fill the PROMs questionnaire, and this effect may differ between patients who live alone and those who do not. The PROMs data contain a question on whether the patient received some assistance in filling the questionnaire. We have therefore performed an additional analysis where we regress a dummy variable related to assistance (=1 if “the patient has received any assistance in the completion of the form”, and =0 if not) on pre-operative health and pre-operative health interacted with living alone, and on the same control variables included in our main model. The results are provided in Table A8. It suggests that patients in poorer health are more likely to receive assistance. For example, in the pre-covid period relative to patients in the group with a 25-32 points pre-operative score, patients with worse health have a higher probability of receiving assistance by 2.6 percentage points for the group with a 17-24 score, by 6.8 pp for the group with a 9-16 score, and 16.2 pp for the group with lowest pre-operative health (0-8 score). However, the interaction of living alone and pre-operative health for groups with a 17-24 score or 9-16 score are statistically insignificant. These two groups account for 65% of the sample. This suggests that patients who live alone are not systematically different for a given level of pre-operative health, and this should reduce concerns about selection effects. The interaction with pre-operative health and living alone is instead statistically significant for the group with 0-8 score, which accounts for 15% of the sample, and equal to 3.7 pp, and the group with a 25-32 score or above 33, though the effect appears quantitative small (less than two pp). Note that in our analysis we already control for pre-operative health and unobserved dimensions of health should be minimal. We have already shown in Table 9 that omitting pre-operative health from the regression does not affect the results on the effect of living alone on length of stay. As an additional robustness check, we have run the main model (Table 2) only on patients with pre-operative health with a score equal to 9-24, where the interaction of living alone with pre-operative health is statistically insignificant in predicting the assistance of filling the form, which cover 65% of the sample. Table A9 suggests that the effect of living alone on length of stay is very similar even when we focus on this sub-sample. This reassures us again that this type of selection is unlikely to affect our results.
